# Exploring Tissue Expander Utility in Kidney Transplant Allograft Nephrectomy

**DOI:** 10.3389/ti.2026.16632

**Published:** 2026-04-20

**Authors:** Ali Hosseinzadeh, Anastasios Giannou, Christopher Nguan, David Harriman

**Affiliations:** 1 Faculty of Medicine, University of British Columbia, Vancouver, BC, Canada; 2 Department of Urologic Sciences, University of British Columbia, Vancouver, BC, Canada

**Keywords:** allograft nephrectomy, end stage kidney disease, kidney transplantation, repeat transplant, tissue expander

Dear Editors,

Kidney transplantation remains the treatment of choice for patients with end-stage renal disease (ESRD), with 5-year graft survival rate of 77%–88% [1, 2]. As patient survival improves, retransplantation now represents a growing proportion of transplant activity. Allograft nephrectomy may be required prior to retransplantation to create space for the subsequent kidney allograft [3, 4]. The procedure is challenging due to dense scarring, distortion of surgical planes, and risk of injury to adjacent vascular, neural, and visceral structures [4]. When retransplantation is performed in the same iliac fossa, surgeons must re-enter a hostile operative field, increasing procedural complexity and risk.

We report a novel application of a breast tissue expander to preserve the iliac fossa following allograft nephrectomy in anticipation of staged kidney retransplantation.

A 37-year-old man with type-1 diabetes and ESRD previously underwent simultaneous kidney-pancreas transplantation, with the pancreas in the right iliac fossa and the renal allograft in the left. Following repeated infections, acute cellular rejection and progressive renal dysfunction, he returned to hemodialysis. After identifying a suitable living donor, we planned to extirpate the failed renal allograft and reimplant the living donor kidney back into the left iliac fossa. Recognizing the technical complexity of allograft nephrectomy and the challenges of reopening a re-scarred fossa for repeat transplantation, the surgical team proposed a novel approach: placement of a tissue expander in the resected fossa after graft removal to preserve space and hopefully limit further scar formation (Figures 1A–C). Following completion of the allograft nephrectomy, a breast tissue expander was soaked in iodine solution, filled with 200 mL of diluted tobramycin and placed into the left iliac fossa. The wound was closed in standard fashion. The patient recovered uneventfully and remained stable during the two-month interval prior to retransplantation.

**FIGURE 1:**
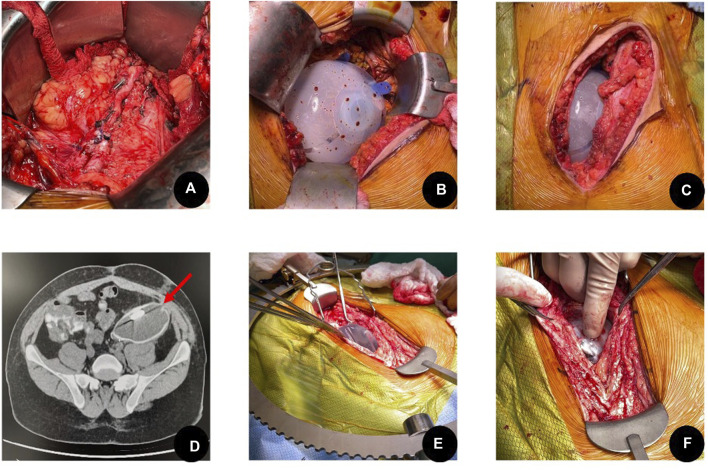
Intraoperative images of the insertion and removal of a breast tissue expander in the left iliac fossa. **(A)** Completed allograft nephrectomy in the left iliac fossa, highlighting the pelvic vessels ready for repeat transplant procedure, **(B)** Insertion of the tissue expander and appropriate sizing with instillation fluid, **(C)** Closure of allograft nephrectomy wound around tissue expander, **(D)** Pre-transplant CT scan demonstrating the implantation of the tissue expander in the left iliac fossa. **(E,F)** Removal of the tissue expander during the retransplantation procedure, highlighting the pseudocapsule created and ease of extraction.

At the time of living-donor transplantation, the expander was encountered within a well-formed pseudocapsule. Extraction was straightforward, and the capsule provided a smooth-walled cavity corresponding to the prior graft bed (Figures 1D-F). Notably, the external iliac vessels and bladder were situated outside the borders of the expander and pseudocapsule, necessitating careful posterior dissection through residual scar tissue to obtain access to the vasculature and bladder. The donor kidney was anastomosed to the external iliac vessels in standard fashion. Immediate graft perfusion and urine output were observed following clamp release. The ureteroneocystostomy was completed over a stent without complication. The patient demonstrated immediate graft function and was discharged on postoperative day seven with stable renal function.

Allograft nephrectomy may be required in cases of failed kidney allograft to create space for subsequent retransplantation, either as a staged procedure or performed simultaneously at the time of retransplantation [5]. The operation is often challenging due to scarring and inflammation around the failed graft, which can increase the risk of increased blood loss, injury to muscle, nerve, vasculature and intraabdominal structures [5]. To our knowledge, this is the first case report describing the use of a tissue expander to limit excessive scarring in the iliac fossa and facilitate subsequent dissection for repeat kidney transplantation on the same operative side. While the tissue expander improved access to the implant bed, the pseudocapsule complicated dissection of the pelvic vessels and bladder, which lay outside it.

The choice between simultaneous or staged nephrectomy during kidney retransplantation remains debated. Simultaneous explant and retransplantation has the advantage of minimizing the number of surgeries and avoiding the anephric state [6]. However, this procedure can result in prolonged anesthesia time, increased risk of intraoperative complications and potentially poor hemodynamic states to support kidney retransplantation [6]. The practice at our center is to stage an allograft nephrectomy, when required, two to three months before repeat transplantation. We found that implantation of the tissue expander in the excised space can potentially redirect scar formation to the periphery of the operative field preserving iliac fossa space. Prolonged implantation of tissue expanders is not a new concept in breast reconstructive surgery, as they are often left *in situ* for between 2–12 months [7]. However, longer periods of implantation are associated with increased scar tissue formation and risk of tissue expander rupture [7]. Immunocompromised patients are at a higher risk of post-operative infection after tissue expander implantation, and in our center all patients considered for retransplantation remain on their maintenance immunosuppression following allograft nephrectomy. In this case, we thoroughly bathed the tissue expander with iodine solution, filled it with diluted tobramycin and gave IV prophylactic meropenem and micafungin at the time of surgery to minimize the risk of bacterial and fungal infection throughout this patient’s course given their past history of infection.

The formation of the pseudocapsule is consistent with previous studies on breast tissue expanders, where capsular contracture is a well-documented phenomenon [8]. The presence of scar tissue around the vessels and bladder underscores the complexity of the fibrotic response. Strategies aimed at reducing pseudocapsule formation, such as antifibrotic agents, may help mitigate scarring and facilitate future dissection, potentially increasing the appeal of tissue expander use. However, the challenges posed by the pseudocapsule were significant enough that our group will likely avoid using tissue expanders in this patient population unless effective strategies to minimize pseudocapsular formation are available.

This case demonstrates a novel application of a tissue expander to preserve iliac fossa space in kidney retransplantation. Although limited to a single case, the technique was feasible and safe, resulting in successful retransplantation with immediate graft function. The pseudocapsule, while helpful in maintaining iliac fossa space, complicated dissection of the iliac vessels and bladder, limiting the broader applicability of this approach at present.

## Data Availability

The raw data supporting the conclusions of this article will be made available by the authors, without undue reservation.
